# Activation of the Carboxypeptidase U (CPU, TAFIa, CPB2) System in Patients with SARS-CoV-2 Infection Could Contribute to COVID-19 Hypofibrinolytic State and Disease Severity Prognosis

**DOI:** 10.3390/jcm11061494

**Published:** 2022-03-09

**Authors:** Karen Claesen, Yani Sim, An Bracke, Michelle De bruyn, Emilie De Hert, Gwendolyn Vliegen, An Hotterbeekx, Alexandra Vujkovic, Lida van Petersen, Fien H. R. De Winter, Isabel Brosius, Caroline Theunissen, Sabrina van Ierssel, Maartje van Frankenhuijsen, Erika Vlieghe, Koen Vercauteren, Samir Kumar-Singh, Ingrid De Meester, Dirk Hendriks

**Affiliations:** 1 Laboratory of Medical Biochemistry, Department of Pharmaceutical Sciences, University of Antwerp, 2610 Wilrijk, Belgium; karen.claesen@uantwerpen.be (K.C.); yani.sim@uantwerpen.be (Y.S.); an.bracke@uantwerpen.be (A.B.); michelle.debruyn2@uantwerpen.be (M.D.b.); emilie.dehert@uantwerpen.be (E.D.H.); gwendolyn.vliegen@histogenex.com (G.V.); ingrid.demeester@uantwerpen.be (I.D.M.); 2 Molecular Pathology Group, Laboratory of Cell Biology & Histology, Faculty of Medical & Health Sciences, University of Antwerp, 2610 Wilrijk, Belgium; an.hotterbeekx@uantwerpen.be (A.H.); fien.dewinter@uantwerpen.be (F.H.R.D.W.); samir.kumar-singh@uantwerpen.be (S.K.-S.); 3 Clinical Virology Unit, Institute of Tropical Medicine, 2000 Antwerp, Belgium; avujkovic@itg.be (A.V.); kvercauteren@itg.be (K.V.); 4 Department of Clinical Sciences, Institute of Tropical Medicine, 2000 Antwerp, Belgium; lvanpetersen@itg.be (L.v.P.); ibrosius@itg.be (I.B.); ctheunissen@itg.be (C.T.); mvanfrankenhuijsen@itg.be (M.v.F.); 5 Department of General Internal Medicine, Infectious Diseases and Tropical Medicine, University Hospital Antwerp, 2650 Edegem, Belgium; sabrina.vanierssel@uza.be (S.v.I.); erika.vlieghe@uza.be (E.V.)

**Keywords:** carboxypeptidase B2, carboxypeptidase U, coronavirus, COVID-19, thrombin-activatable fibrinolysis inhibitor

## Abstract

Coronavirus disease 2019 (COVID-19) is a viral lower respiratory tract infection caused by the highly transmissible and pathogenic SARS-CoV-2 (severe acute respiratory-syndrome coronavirus-2). Besides respiratory failure, systemic thromboembolic complications are frequent in COVID-19 patients and suggested to be the result of a dysregulation of the hemostatic balance. Although several markers of coagulation and fibrinolysis have been studied extensively, little is known about the effect of SARS-CoV-2 infection on the potent antifibrinolytic enzyme carboxypeptidase U (CPU). Blood was collected longitudinally from 56 hospitalized COVID-19 patients and 32 healthy controls. Procarboxypeptidase U (proCPU) levels and total active and inactivated CPU (CPU+CPUi) antigen levels were measured. At study inclusion (shortly after hospital admission), proCPU levels were significantly lower and CPU+CPUi antigen levels significantly higher in COVID-19 patients compared to controls. Both proCPU and CPU+CPUi antigen levels showed a subsequent progressive increase in these patients. Hereafter, proCPU levels decreased and patients were, at discharge, comparable to the controls. CPU+CPUi antigen levels at discharge were still higher compared to controls. Baseline CPU+CPUi antigen levels (shortly after hospital admission) correlated with disease severity and the duration of hospitalization. In conclusion, CPU generation with concomitant proCPU consumption during early SARS-CoV-2 infection will (at least partly) contribute to the hypofibrinolytic state observed in COVID-19 patients, thus enlarging their risk for thrombosis. Moreover, given the association between CPU+CPUi antigen levels and both disease severity and duration of hospitalization, this parameter may be a potential biomarker with prognostic value in SARS-CoV-2 infection.

## 1. Introduction

Coronavirus disease 2019 (COVID-19) is a viral lower respiratory tract infection caused by the highly transmissible and pathogenic SARS-CoV-2 (severe acute respiratory syndrome coronavirus-2). The infection primarily affects the respiratory system with the majority of individuals with SARS-CoV-2 developing no or minimal symptoms (including fever, cough, myalgia, headache, and taste and smell dysfunction) [1,2]. About 15% of patients develop viral pneumonia with significant hypoxemia that requires oxygen support. In approximately 5% of patients, this hypoxemic respiratory failure leads to a rapidly evolving severe acute respiratory distress syndrome (ARDS), sepsis, and/or multiorgan failure [3,4,5].

Besides respiratory failure, systemic thromboembolic complications are frequent in COVID-19 patients and the result of a dysregulation of the hemostatic balance [2,6,7,8,9]. Abnormalities observed in most patients include a (minimally) prolonged prothrombin time, low antithrombin concentrations, elevated fibrinogen, and mild (if any) thrombocytopenia [3,6,10,11]. Findings of elevated PAI-1 levels and substantially reduced clot lysis as measured by thromboelastography point towards hypofibrinolysis in COVID-19 patients [7,8,12,13]. Moreover, markedly increased plasma D-dimer concentrations are a hallmark of COVID-19 and strongly suggestive for plasmin-mediated fibrinolysis following activation of the coagulation cascade [6,11,14]. COVID-19 thus causes the hemostatic balance to tip towards an overall hypercoagulable and hypofibrinolytic state.

The enzyme carboxypeptidase U (CPU, CPB2, TAFIa) is a potent inhibitor of fibrinolysis. After activation of the zymogen procarboxypeptidase U (proCPU, proCPB2, TAFI) by thrombin (-thrombomodulin) or plasmin, CPU delays efficient plasminogen activation by cleaving off C-terminal lysines from partially degraded fibrin [15,16,17]. As such, CPU counteracts the progression of fibrinolysis. The question that arises is whether CPU may play a role in the hypofibrinolytic state observed in COVID-19 patients and, thus, may contribute to the high prothrombotic status in these patients. Here, we explored the effect of SARS-CoV-2 infection on the CPU system by measuring both proCPU and CPU+CPUi over time.

## 2. Materials and Methods

### 2.1. Study Design and Participants

This was a post hoc analysis within the COVID-19 Immune Repertoire Sequencing (IMSEQ) study, a single-center prospective cohort study conducted at the Antwerp University Hospital (UZA) of which the study design was previously described (clinical trials.gov NCT04368143) [18]. The study was approved by the institutional Ethics Committee UZA/UAntwerp (20/12/135) and the ITM IRB, and written informed consent was obtained from all participants or their legal representative at inclusion.

Patients (≥18 years of age) with SARS-CoV-2 infection (confirmed by SARS-CoV-2 polymerase chain reaction (PCR) test) were included shortly after hospital admission. Clinical data and recorded interventions (e.g., routine laboratory parameters, use of antivirals, antibiotics, respiratory support, etc.) were collected from the electronic patient medical file. Disease severity was assessed by the WHO COVID-19 disease severity categorization [1].

Clinically healthy individuals were recruited at the Institute of Tropical Medicine Antwerp as a control population for the comparison of CPU-related parameters. Some of these individuals previously tested positive for SARS-CoV-2 (COVID-19 exposed controls), while others did not have any evidence of SARS-CoV-2 exposure (COVID-19 non-exposed controls).

### 2.2. Sample Collection and Biochemical Analyses

Blood samples were collected longitudinally from hospitalized COVID-19 patients: the inclusion time point was shortly after hospital admission, with follow-up time points until discharge. In control subjects, two blood collections were performed approximately four weeks apart. Blood was drawn in lithium heparin tubes (16 IU/mL blood, S-Monovette^®^, Nümbrecht, Germany). Plasma proCPU concentrations were determined as previously described [19,20]. Antigen levels of activated and inactivated CPU (CPU+CPUi) were measured by ELISA according to the manufacturer’s instructions (Asserachrom TAFIa/TAFIai, Diagnostica Stago, Asnières-sur-Seine, France), and routine laboratory parameters of the COVID-19 cohort were determined at the hospital’s clinical laboratory.

### 2.3. Statistical Analysis

Results were expressed as mean ± standard deviation (SD). GraphPad Prism version 9 was used for statistical analysis and data plotting. A Kolmogorov–Smirnov test showed that none of the continuous variables were normally distributed. A Mann–Whitney U test (unpaired data) or a Wilcoxon matched-pairs signed rank test (paired data) were performed to compare differences between continuous variables. Spearman correlation coefficients were computed to assess possible associations. A *p*-value < 0.05 was considered statistically significant.

## 3. Results and Discussion

### 3.1. Patient Characteristics

Patient enrollment took place at the Antwerp University Hospital between April 2020 and February 2021. During the study period, a total of 56 patients (38 male, 18 female) with laboratory-confirmed COVID-19 were recruited. The mean age was 58 ± 14 years (range 29–84 years). On average, patients with SARS-CoV-2 infection were hospitalized for 19 ± 6 days (range 3–61 days) in this study. Additional characteristics of the COVID-19 patients are summarized in Table 1.

For the comparison of CPU-related parameters, 32 clinically healthy subjects were included (21 male, 11 female; mean age 45 ± 12 years (range 19–62 years)). Although the control group was younger on average, the age difference with the COVID-19 group was not statistically significant. Of those 32 individuals, 14 had a history of a PCR-confirmed SARS-CoV-2 infection (at least 2 months before inclusion) or a positive serological test result (COVID-19 exposed controls; 11 male, 3 female; mean age 41 ± 15 years (range 19–62 years)), while the other 18 did not have any indications of a SARS-CoV-2 infection history (i.e., no known high-risk contact and absence of COVID-19 clinical symptoms, or a negative PCR test in the event of a high-risk contact or COVID-19 clinical symptoms) (COVID-19 non-exposed controls; 10 male, 8 female; mean age 45 ± 11 years (range 24–59 years)).

### 3.2. ProCPU and CPU+CPUi Antigen Levels Do Not Differ between COVID-19 Exposed and Non-Exposed Controls

**Figure 1:**
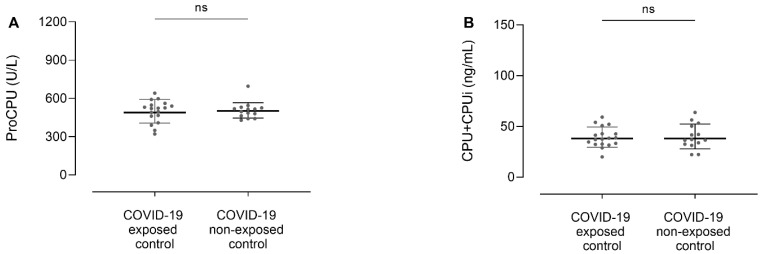
Plasma procarboxypeptidase U (proCPU) levels (**A**) and total active and inactivated carboxypeptidase U (CPU+CPUi) antigen levels (**B**) in COVID-19 exposed (N = 14) and COVID-19 non-exposed (N = 18) controls. COVID-19 exposed controls are individuals that previously tested positive for SARS-CoV-2 (PCR-confirmed SARS-CoV-2 infection (at least 2 months before inclusion) or a positive serological test result), while COVID-19 non-exposed controls are individuals without any evidence of SARS-CoV-2 exposure. Data are presented as mean ± SD. Mann–Whitney U test; ns = not significant.

To evaluate whether there was a long-term effect of COVID-19 on CPU-related parameters in clinically healthy individuals that had previously experienced the disease, the proCPU and CPU+CPUi antigen levels of the COVID-19 exposed controls were compared with those of the COVID-19 non-exposed controls. Neither proCPU (*p* = 0.66) (Figure 1A) nor CPU+CPUi antigen levels (*p* = 0.96) (Figure 1B) significantly differed between the two control groups. In further analysis, COVID-19 exposed and non-exposed controls were merged into a single control group.

### 3.3. ProCPU Consumption with Concomitant CPU Generation in COVID-19 Patients upon Hospital Admission

ProCPU and CPU+CPUi antigen levels were measured in longitudinal COVID-19 patient samples. CPU+CPUi measurements are a good indicator of recent and ongoing CPU activation, representing the total amount of active CPU and thermally inactivated CPU (CPUi) present at a certain time point.

**Figure 2:**
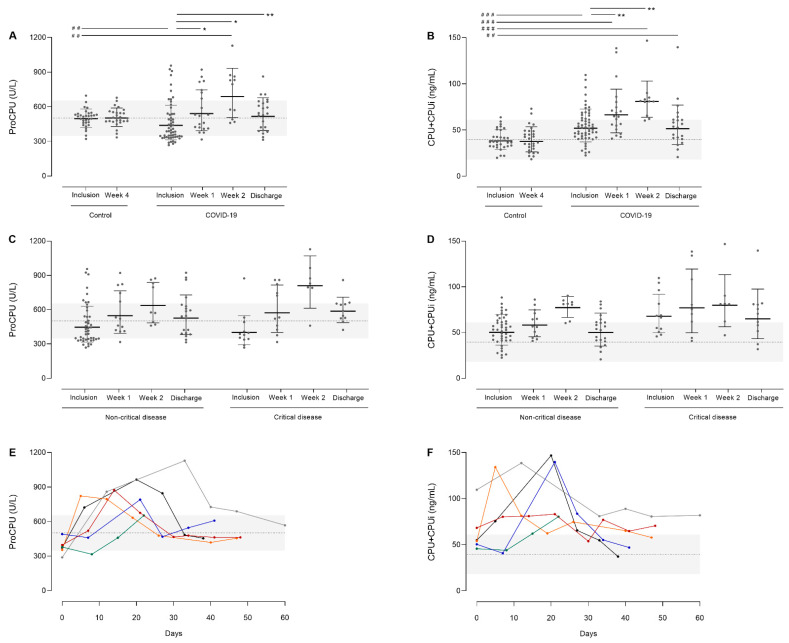
(**A**,**B**) Time course of plasma procarboxypeptidase U (proCPU) levels (**A**) and total active and inactivated carboxypeptidase U (CPU+CPUi) antigen levels (**B**) in hospitalized COVID-19 patients (N = 12–56) at inclusion (ranging from 1–5 days after hospital admission), 1 week after inclusion (ranging from 5–8 days after inclusion), 2 weeks after inclusion (ranging from 12–15 days after inclusion), and at discharge (ranging from 17–61 days after inclusion), and in clinically healthy controls at inclusion and 28 days later (N = 32). Data are presented as mean ± SD. Mann–Whitney U test (unpaired data); ## *p* < 0.01; and ### *p* < 0.001. Wilcoxon Matched-Pairs Signed Rank test (paired data); * *p* < 0.05; ** *p* < 0.01. The horizontal dotted line represents the mean proCPU level or CPU+CPUi antigen level of the healthy controls with the corresponding confidence interval (2*SD; grey area). (**C**,**D**) Time course of plasma proCPU levels (**C**) and CPU+CPUi antigen levels (**D**) at inclusion (ranging from 1–5 days after hospital admission), 1 week after inclusion (ranging from 5–8 days after inclusion), 2 weeks after inclusion (ranging from 12–15 days after inclusion), and at discharge (ranging from 17 to 61 days after inclusion) in COVID-19 patients with critical disease (right; N = 12) versus non-critical disease (left; N = 44). Data are presented as mean ± SD. The horizontal dotted line represents the mean proCPU level or CPU+CPUi antigen level of the healthy controls with the corresponding confidence interval (2*SD; grey area). Mann–Whitney U test (unpaired data); # *p* < 0.05; ## *p* < 0.01; and ### *p* < 0.001. Wilcoxon Matched-Pairs Signed Rank test (paired data); * *p* < 0.05; ** *p* < 0.01; *** *p* < 0.001. (**E**,**F**) Individual proCPU (**E**) and CPU+CPUi antigen level (**F**) profiles of six critically ill patients (samples at ≥4 time points available). The horizontal dotted line represents the mean proCPU level or CPU+CPUi antigen level of the healthy controls with the corresponding confidence interval (2*SD; grey area).

Shortly after hospital admission (inclusion time point), mean proCPU levels were significantly lower in COVID-19 patients (466 ± 130 U/L) compared to controls (501 ± 77 U/L; *p* = 0.01) (Figure 2A). Lower proCPU levels in COVID-19 patients (N = 14) compared to healthy controls (N = 14) were also reported by Juneja and co-workers [21]. Mean CPU+CPUi antigen levels on the other hand were significantly higher in COVID-19 patients shortly after hospital admission (54.9 ± 17.8 ng/mL vs. 41.2 ± 23.6 ng/mL; *p* < 0.0001) (Figure 2B). These results reflect the consumption of proCPU and thus the ongoing cleavage of proCPU with concomitant CPU generation in the early phase of SARS-CoV-2 infection. An increase in active CPU is known to slow down fibrinolysis, and thus can contribute to the hypofibrinolytic status of COVID-19 patients and will likely enlarge the risk of thrombosis in these patients.

### 3.4. Time Course of CPU-Related Parameters

To get a more complete picture of the effect of SARS-CoV-2 infection on the CPU system, proCPU and CPU+CPUi antigen levels were further measured during patient hospitalization.

#### 3.4.1. Total Study Population

Following low proCPU concentrations early after admission (inclusion time point), a pronounced elevation of proCPU levels was observed in COVID-19 patients during the first week. Around day 14, proCPU levels were significantly higher compared to controls (*p* < 0.001) and to initial values (*p* = 0.002) (Figure 2A). Hereafter, proCPU levels declined, with levels at discharge that were comparable to those of the controls (*p* = 0.99) (Figure 2A). ProCPU levels in the control cohort remained stable over a period of 28 days (*p* = 0.99). (Figure 2A). 

In addition, measurement of CPU+CPUi antigen levels over time showed that these levels also progressively increased in COVID-19 patients up to approximately day 14. This period was followed by a clear and significant decrease in CPU+CPUi antigen levels, with CPU+CPUi antigen levels at discharge similar to those shortly after admission. Compared to controls, CPU+CPUi antigen levels at discharge were still elevated in COVID-19 patients (Figure 2B). CPU+CPUi antigen levels of the control cohort remained stable over a period of 28 days (*p* = 0.93) (Figure 2B). The time course of these parameters shed light on the changes in CPU-related parameters caused by COVID-19. Most likely, the hypercoagulable state in SARS-CoV-2 infection results in elevated concentrations of thrombin, which will cause increased proCPU consumption (evidenced by a decrease in proCPU levels shortly after admission) together with CPU generation, and explains the rise in CPU+CPUi antigen levels over time. The activation of the CPU system arising from an inflammation-driven increase in thrombin concentration is not only seen in COVID-19, but also in other inflammatory disease states which is supported by several studies investigating the CPU system in sepsis. In these studies, admission proCPU levels were consistently decreased in septic patients compared to controls, while CPU+CPUi antigen levels were significantly higher at that time [22,23,24,25,26,27]. Moreover, CPU+CPUi antigen levels were significantly higher in non-survivors versus survivors and strongly correlated with the severity scores of the disease [27,28,29]. The secondary increase in proCPU concentrations on the other hand is most likely the result of an increase in proCPU synthesis in the liver. Interestingly, Lustenberger and colleagues described a similar time course for proCPU in trauma-induced coagulopathy, including a secondary increment in proCPU levels as observed here in the setting of COVID-19 [30] A possible explanation for this increased proCPU synthesis is that the inflammatory environment in COVID-19 causes a proCPU upregulation since it is known that plasma proCPU concentrations are subject to inflammation and that inflammatory cytokines are able to modulate the expression of *CPB2*, the gene encoding proCPU [30,31].

#### 3.4.2. Critical versus Non-Critical Disease

When comparing the time course of proCPU levels in COVID-19 patients with critical vs. non-critical disease, the above-described pattern of low admission proCPU levels that markedly increased during the first weeks of hospitalization, and an overall normalization at discharge were clearly visible in both groups (Figure 2C). While the secondary rise in proCPU levels seems to be more pronounced in the critical disease cohort, no statistical significance was reached comparing patients with non-critical vs. critical disease at any time point. The detailed individual proCPU profiles of six critically ill patients (patients with ≥4 sampling time points available) also showed a similar time course (Figure 2E).

The time courses of CPU+CPUi antigen levels in the critical and non-critical disease cohort were also comparable and followed the earlier described trend of elevated admission levels that further increased up to approximately day 14 and normalized at discharge. However, the normalization of the CPU+CPUi antigen levels is slower in patients with critical disease (Figure 2D). Moreover, the detailed individual CPU+CPUi antigen level profiles of the same six critically ill patients showed substantial inter-individual variability in CPU+CPUi antigen levels (Figure 2F). While two out of six patients displayed only limited CPU activation (CPU+CPUi peak levels < 70 ng/mL), the other four displayed extensive CPU activation (>130 ng/mL). In the latter patients, the extent of CPU+CPUi peak levels was similar, but the time when these peak levels were reached, was highly variable among patients and ranged between 5 and 22 days.

### 3.5. C-Reactive Protein (CRP) Levels Correlate with the Decrease in ProCPU Levels Early after Disease Onset

**Figure 3:**
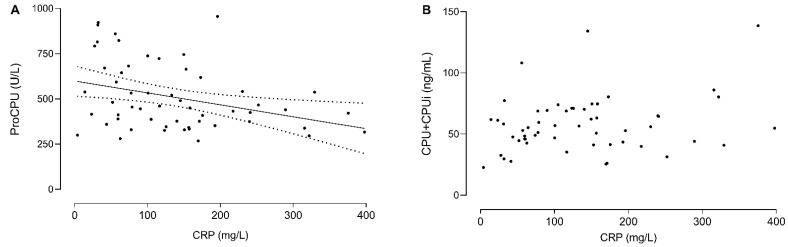
Relationship between the highest recorded CRP levels and corresponding proCPU levels (**A**) or CPU+CPUi antigen levels (**B**). For the majority of the patients, the highest CRP value was measured within one week after enrolment in the study. Spearman correlation coefficient r was determined for both correlations. In case of a significant correlation (*p* > 0.05), linear regression analysis was performed, and the best-fit line (solid line) with 95% confidence bands was plotted (dashed lines).

To better understand the link between inflammation and CPU activation/proCPU consumption in SARS-CoV-2 infection, the relationship between the highest recorded CRP value and the corresponding proCPU concentrations or CPU+CPUi antigen level was assessed. For the majority of the patients, the highest CRP value was measured within one week after enrolment in the study. A negative correlation between the highest recorded CRP levels and the corresponding proCPU levels was observed (r = −0.43, *p* = 0.015) (Figure 3A): the highest CRP values were seen in patients with the largest initial proCPU reduction. CPU+CPUi antigen levels at the same time point did not however correlate with these CRP levels (r = 0.14, *p* = 0.28) (Figure 3B).

Thus, there is a correlation between the ongoing inflammation in SARS-CoV-2 infection and the decrease in proCPU early after the onset of the disease. For CPU+CPUi antigen levels, such a correlation was not observed. This may be related to CPU+CPUi kinetics, but knowledge about this is limited, especially regarding CPUi kinetics.

### 3.6. Baseline CPU+CPUi Antigen Levels Correlate with Disease Severity and the Duration of Hospitalization

**Figure 4:**
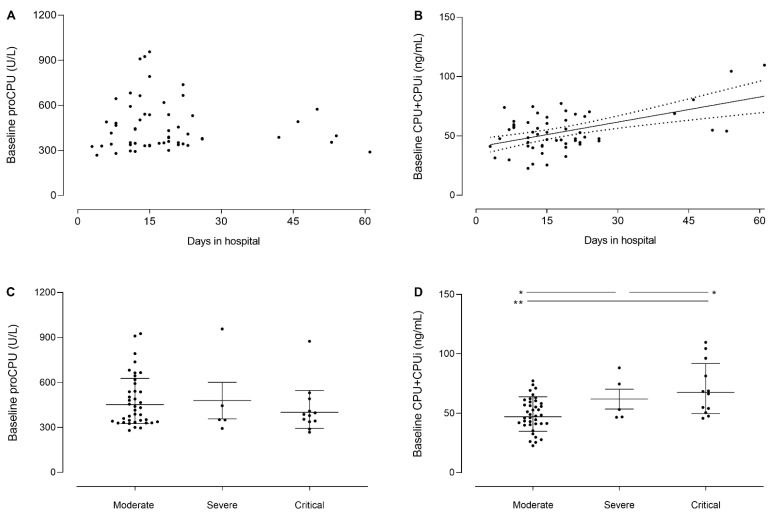
Correlation between the duration of hospitalization and both baseline (inclusion time point shortly after hospital admission) procarboxypeptidase U (proCPU) (**A**) and total active and inactivated carboxypeptidase U (CPU+CPUi) antigen levels (**B**) in hospitalized COVID-19 patients (N = 56). Spearman correlation coefficient r was determined. For statistically significant correlations (*p* < 0.05), linear regression analysis was performed and the best-fit line (solid line) with 95% confidence bands was plotted (dashed lines). Baseline proCPU levels (**C**) and baseline CPU+CPUi antigen levels (**D**) of hospitalized COVID-19 patients grouped by disease severity (WHO COVID-19 disease severity categorization): moderate (N = 39), severe (N = 5), and critical (N = 12). Mann–Whitney U test (unpaired data); * *p* < 0.05; ** *p* < 0.01.

Possible associations between CPU-related parameters and both disease severity and the duration of hospitalization were investigated. Baseline CPU+CPUi antigen levels, but not proCPU levels, were found to be positively correlated with the duration of a patient’s hospital stay (r = 0.53; *p* < 0.001) (Figure 4A,B). Further, when plotting admission levels of these parameters stratified by disease severity (moderate, severe, and critical), it is apparent that CPU+CPUi antigen levels shortly after admission are positively related to disease severity (sicker patients present with higher CPU+CPUi antigen levels), while no relationship with disease severity was observed for proCPU levels (Figure 4C,D). Accordingly, Nougier and co-workers described significantly higher CPU+CPUi antigen levels at admission in 48 ICU versus 30 non-ICU COVID-19 patients [6].

The association of CPU+CPUi antigen levels with both disease severity and the duration of hospitalization suggests that this parameter may be a potential biomarker with prognostic value and supports further studies on CPU+CPUi antigen levels, as well as CPU activity in the context of SARS-CoV-2 infection.

Because of the focus of this study on the CPU system and its related parameters, additional hemostasis parameters were not followed in detail over time. Moreover, no group of patients with asymptomatic SARS-CoV-2 infection was included here; therefore, it is unclear how the current results will generalize in this type of patient population. Future research should profile the changes in CPU-related parameters to answer this. In addition, future work should also focus on possible correlations of fibrinolytic activity, and CPU-related parameters in particular, with thrombotic complications in patients with severe SARS-CoV-2 infection. This was not possible in the current study, given that none of the COVID-19 patients enrolled in our study had clinical evidence for thrombosis and that systematic screening for thrombotic complications with ultrasound and computed tomography with pulmonary angiography (CTPA) were not included in the standard care [32]. Moreover, the sample size of our study was not calculated to allow the evaluation of this correlation [33]. Finally, given the similar/parallel observations in COVID-19 and sepsis with regard to the activation of the CPU system, future research examining the effect of COVID-19 and sepsis on this pathway side by side is of interest. This will not only advance our understanding of common and divergent aspects of this pathway during COVID-19 and sepsis, but also broaden our knowledge on the activation and role of the CPU system in diseases with an inflammatory basis in general.

## 4. Conclusions

In conclusion, we showed initial significant generation of CPU with concomitant proCPU consumption during the early phase of SARS-CoV-2 infection, with a subsequent progressive increase in both proCPU concentrations and CPU+CPUi antigen levels in hospitalized COVID-19 patients. These alterations in CPU-related parameters will (at least partly) contribute to the fibrinolysis shutdown observed in COVID-19 patients and are likely to enlarge their risk of thrombosis. These results point to the potential of CPU inhibitors to be used as therapeutic agents in COVID-19 patients in the future to enhance these patients’ fibrinolytic capacity. However, further studies are needed on this topic. Moreover, CPU+CPUi antigen levels around admission were related to disease severity and the duration of hospitalization, putting forward the hypothesis that high circulating CPU+CPUi antigen levels may be a potential biomarker with prognostic value in SARS-CoV-2 infection. 

## Figures and Tables

**Table 1:** Clinical and biological characteristics of COVID-19 patients (N = 56).

**Demographics**		
Age—years (range)	58 (29–84)	
Sex		
Male—N (%)	38 (68%)	
Female—N (%)	18 (32%)	
**Baseline Clinical Parameters**		
Comorbidities		
Obesity	13 (22%)	
Diabetes	9 (16%)	
Chronic respiratory disease	10 (17%)	
Cardiovascular disease	10 (17%)	
Cancer	6 (10%)	
SpO2 at admission (%)	96 ± 4	
WHO severity classification		
Moderate	39 (70%)	
Severe	5 (9%)	
Critical	12 (21%)	
**Laboratory Parameters**		**Reference Value**
Platelet count (×10^9^/L)	181 ± 79	166–396
WBC (×10^9^/L)	8.6 ± 6.0	4.2–10.3
CRP (mg/L)	94 ± 125	<10
**Hospital Care**		
Medication use		
Antibiotics	36 (62%)	
Antivirals	4 (7%)	
Antifungals	1 (2%)	
Steroids	25 (43%)	
Vasoactive medications	7 (12%)	
Antiplatelet agent	2 (3%)	
Anticoagulation	8 (14%)	
Respiratory status		
Room air	6 (10%)	
High-flow nasal oxygen	44 (76%)	
Invasive ventilation	7 (12%)	
Extracorporeal life support	1 (2%)	
**Outcome**		
ICU stay	9 (16%)	
Days in hospital	19 ± 6	
In-hospital death	4 (7%)	

Note: Results are given as a number (N) with a percentage in parentheses or as mean ± standard deviation (SD). Abbreviations: COVID-19, coronavirus disease 19; CRP, C-reactive protein; ICU, intensive care unit; and WBC, white blood cell.

## Data Availability

The data presented in this study are available on request from the corresponding author.
